# Stimulated Immune Response by TruCulture^®^ Whole Blood Assay in Patients With European Lyme Neuroborreliosis: A Prospective Cohort Study

**DOI:** 10.3389/fcimb.2021.666037

**Published:** 2021-05-10

**Authors:** Mathilde Ørbæk, Rosa Maja Møhring Gynthersen, Helene Mens, Christian Stenør, Lothar Wiese, Christian Brandt, Sisse Rye Ostrowski, Susanne Dam Nielsen, Anne-Mette Lebech

**Affiliations:** ^1^ Department of Infectious Diseases, Copenhagen University Hospital, Rigshospitalet, Copenhagen, Denmark; ^2^ Cluster for Molecular Imaging, Department of Biomedical Sciences, University of Copenhagen, Copenhagen, Denmark; ^3^ Department of Neurology, Herlev Hospital, Herlev, Denmark; ^4^ Department of Clinical Medicine, Faculty of Health and Medical Sciences, University of Copenhagen, Copenhagen, Denmark; ^5^ Department of Infectious Diseases, Sjællands University Hospital, Roskilde, Denmark; ^6^ Department of Clinical Immunology, Copenhagen University Hospital, Rigshospitalet, Copenhagen, Denmark

**Keywords:** stimulated immune response, TruCulture, whole blood assay, Lyme neuroborreliosis, *Borrelia burgdorferi* sensu lato complex, innate immune system

## Abstract

**Introduction:**

*Borrelia burgdorferi* sensu lato complex (*B. burgdorferi*) can cause a variety of clinical manifestations including Lyme neuroborreliosis. Following the tick-borne transmission, *B. burgdorferi* initially evade immune responses, later symptomatic infection is associated with occurrence of specific antibody responses. We hypothesized that *B. burgdorferi* induce immune hyporesponsiveness or immune suppression and aimed to investigate patients with Lyme neuroborreliosis ability to respond to immune stimulation.

**Methods:**

An observational cohort study investigating the stimulated immune response by standardized whole blood assay (TruCulture^®^) in adult patients with Lyme neuroborreliosis included at time of diagnosis from 01.09.2018-31.07.2020. Reference intervals were based on a 5-95% range of cytokine concentrations from healthy individuals (n = 32). Patients with Lyme neuroborreliosis and references were compared using Mann-Whitney U test. Heatmaps of cytokine responses were generated using the webtool Clustvis.

**Results:**

In total, 22 patients with Lyme neuroborreliosis (19 definite, 3 probable) were included. In the unstimulated samples, the concentrations of cytokines in patients with Lyme neuroborreliosis were comparable with references, except interferon (IFN)-α, interleukin (IL)-17A, IL-1β and IL-8, which were all significantly below the references. Patients with Lyme neuroborreliosis had similar concentrations of most cytokines in all stimulations compared with references. IFN-α, IFN-γ, IL-12 and IL-17A were lower than references in multiple stimulations.

**Conclusion:**

In this exploratory cohort study, we found lower or similar concentrations of circulating cytokines in blood from patients with Lyme neuroborreliosis at time of diagnosis compared with references. The stimulated cytokine release in blood from patients with Lyme neuroborreliosis was in general slightly lower than in the references. Specific patterns of low IL-12 and IFN-γ indicated low Th1-response and low concentrations of IL-17A did not support a strong Th17 response. Our results suggest that patients with Lyme neuroborreliosis elicit a slightly suppressed or impaired immune response for the investigated stimulations, however, whether the response normalizes remains unanswered.

## Introduction

Lyme borreliosis (LB) is a tick-borne infection caused by the *Borrelia burgdorferi* sensu lato complex (*B. burgdorferi*). *B. burgdorferi* can cause a variety of clinical manifestations and without treatment the infection can disseminate to various tissues, causing Lyme arthritis, Lyme carditis, acrodermatitis chronica atrophicans or Lyme neuroborreliosis (LNB) (Stanek et al., 2012; Hansen et al., 2013). The *B. burgdorferi* sensu lato complex now comprises of more than 20 different genospecies (Waindok et al., 2017; Cutler et al., 2019; Margos et al., 2020) of which the genospecies *B. burgdorferi* sensu stricto, *B. afzelii* and *B. garinii* primarily are responsible for human disease (Stanek et al., 2012; Waindok et al., 2017).

Following the transmission of *B. burgdorferi*, the spirochete encounters many levels of host defense (Embers et al., 2004; Petzke and Schwartz, 2015). Early on, the transmission is enhanced by several tick salivary proteins, which evade the immunologic recognition system (Shemenski, 2019). Furthermore, *B. burgdorferi* expresses a variety of outer surface proteins (Osp), which modulate the host defense system and both the innate and the adaptive immune responses (Embers et al., 2004; Petzke and Schwartz, 2015; Oosting et al., 2016). The innate immune response directs the adaptive immune response and is sustained until the adaptive immune system is able to control the infection (Petzke and Schwartz, 2015). Relatively little is known about the innate immune receptors mediating the inflammatory response to *B. burgdorferi* infection (Petzke et al., 2009). However, when a pathogen enters the human body, a cascade of recognition molecules is activated by host cellular receptors, including toll-like receptors (TLRs) (Petzke et al., 2009), leading to the production of inflammatory mediators i.e. chemokines and cytokines causing much of the pathology to LNB (Sjöwall et al., 2005; Petnicki-Ocwieja and Kern, 2014). The TLRs known to be involved in *B. burgdorferi* infection are primarily TLR2, which binds to Osps in heterodimers with TLR1 or 6 (Ozinsky et al., 2000; Oosting et al., 2010; Petnicki-Ocwieja and Kern, 2014). Furthermore, TLR8 seems to be able to recognize *B. burgdorferi* RNA after phagocytosis (Cervantes et al., 2013) and though *B. burgdorferi* does not contain lipopolysaccharide (LPS) on its outer surface, an overexpression of TLR4 has been shown after *B. burgdorferi* stimulation (Dudek et al., 2017).

The *B. burgdorferi* spirochete is, like syphilis, able to evade the host response and at the same time elicit an adaptive host response leading to high titers of specific antibodies. This antibody response is not sufficient to eradicate the pathogen nor limit dissemination (Diterich et al., 2001; Embers et al., 2004; Pachner and Steiner, 2007). However, over time, most cases of *B. burgdorferi* infection are thought to be self-resolving (Kruger et al., 1989; Steere et al., 2016). We hypothesized that *B. burgdorferi* induces hyporesponsiveness or immune suppression of the host immune system to establish and sustain infection. With the present study, we aimed to investigate patients with LNB’s ability to respond to new immune stimulation.

## Methods

### Study Design and Population

This study is an observational cohort study, investigating the stimulated immune response as a proxy for immune function by a standardized whole blood assay (TruCulture^®^) in blood from adult (≥18 years) patients with LNB. The patients were prospectively enrolled within one day prior to antibiotic initiation for LNB and up to 7 days after ended antibiotic treatment from the Department of Infectious Diseases, Rigshospitalet, Denmark between 1^st^ of September 2018 to 31^st^ of July 2020. Laboratory analyses of blood and CSF, baseline characteristics and clinical findings were retrieved from a quality assessment database. A total of 32 healthy individuals in the working age (≥18-<67 years) were included as an anonymous reference group. For each cytokine, a reference interval was based on the 5-95% range of the cytokine concentration in the reference group. Furthermore, 9 patients with other infections in the central nervous system (CNS). Viral meningitis (n=4), bacterial meningoencephalitis (n=2) and cerebral abscesses (n=3) were included for comparison.

### Definitions

According to the European Federation of Neurological Societies (EFNS) guidelines, LNB was defined by the following three criteria for definite LNB, and two of them for possible LNB: (i) neurological symptoms; (ii) cerebrospinal fluid (CSF) pleocytosis (>5 leucocytes/µL); (iii) *B. burgdorferi* antibodies produced intrathecally (Mygland et al., 2010).

### TruCulture^®^

TruCulture^®^ (Myriad RBM, Austin, USA) reveals the stimulated innate immune response in whole blood by quantifying the release of soluble immune activation products in the supernatant after new stimulation (Duffy et al., 2014; Duffy et al., 2017). TruCulture^®^ consists of four different immune cell stimulations, mimicking the presence of fungal (heat killed *Candida albicans*, HKCA), bacterial (LPS from *Ecsherichia coli*) and two viral agents (Resiquimod R848 and polyinosinic:polycytodylic acid, Poly I:C), to obtain a broad function of different signaling pathways (TLR1/2/4/6, TLR4, TLR7/8 and TLR3, respectively). A fifth tube containing the TruCulture^®^ media without stimulus reveals the *in vivo* activation of blood immune cells and serves as a proxy for circulating cytokine levels. In brief, peripheral blood was collected into lithium heparin anticoagulated whole blood tubes and aliquoted into the prewarmed TruCulture^®^ stimulated or unstimulated tubes after 60 minutes (± 15 min). Subsequently, the TruCulture^®^ tubes were inserted into a digital dry block heater (WWR International A/S, Søborg, Denmark) maintained at 37°C for 22 hours (± 30 min). After incubation supernatants were aliquoted and frozen at -20°C and transferred to -80°C after 1-7 days until thawed for analysis.

The concentrations of following cytokines and chemokines were measured: interleukin (IL-)-1β, IL-6, IL-8, IL-10, IL-12, IL-17A, interferon (IFN)-α, IFN-γ and tumor necrosis factor (TNF)-α by a 8-plex Luminex (LX200, R&D Systems, BIO-Teche LTD) and the results are reported in pg/mL. IFN-α was only available in 20 patients with LNB and 16 references.

### Statistical Analysis

Categorical variables were reported as counts and percentages and continuous variables were summarized as median with interquartile range (IQR) and compared using Mann-Whitney U test. To accommodate concentrations below the lowest measuring point, which transcript to 0, 0.1 was added to all samples on the logarithmic scale. SAS statistical software Enterprise guide version 7.1 (SAS Institute Inc., Cary, NC, USA) was used for data analysis and *p*-values below 0.05 (two-sided) were considered statistically significant. Heatmaps of individual cytokine response signatures were generated using the webtool Clustvis (https://biit.cs.ut.ee/clustvis/) by scaling and centering cytokine concentrations. Column (diagnosis) dendrograms were drawn based on hierarchical clustering analysis using the complete agglomeration method on Euclidian distance matrices and Ward linkage.

## Results

In total, 22 patients with LNB (19 definite, 3 probable) were included, baseline characteristics are listed in **Table 1**.

**Table 1:** Baseline characteristics of 22 patients with Lyme neuroborreliosis at time of first blood sample.

	Lyme neuroborreliosis(n = 22)
Age, median (IQR)	64.5 (61.0-72.4)
Male, n (%)	15 (68)
No comorbidities, n (%)	11 (50)
No prescription medication^1^, n (%)	7 (32)
Clinical presentation	
Duration of symptoms, n (%)	
<6 weeks	13 (59)
6 weeks – 3 months	7 (32)
3 – 6 months	2 (9)
Cranial nerve palsy, n (%)	10 (45)
Sensory nerve palsy, n (%)	11 (50)
Peripheral motor nerve palsy, n (%)	7 (32)
Radiating pain, n (%)	17 (77)
Diagnosis	
Definite LNB	19 (86)
Probable LNB	3 (14)
Laboratory results	
Serum leucocytes, median (IQR)	6.8 (5.8 – 7.7)
Serum neutrophils, median (IQR)	4.0 (3.4 – 5.3)
Serum *B. burgdorferi* IgM and/or IgG, n (%)	
Positive	21 (95)
Not measured	1 (5)
CSF pleocytosis > 5 leucocytes/µL, n (%)	22 (100)
Intrathecal *B. burgdorferi* antibody production IgM/IgG, n (%)	
Positive IgM	14 (65)
Positive IgG	13 (60)
Neither IgM nor IgG	3 (14)
CXCL13 above threshold^2^	5 (23)
Treatment	
Oral Doxycycline	7 (32)
IV Ceftriaxone/Penicillin	7 (32)
IV Ceftriaxone/Penicillin followed by oral Doxycycline	8 (36)
Treatment duration, median (range)	14 (14-21)

IQR, Interquartile range; B. burgdorferi, B. burgdorferi sensu lato complex; Ig, Immunoglobulin; CSF, Cerebrospinal fluid; IV, intravenous.

Categorical variables are presented as n (%) and continuous variables as medians with interquartile rates (IQRs).

^1^Prescriptions of antibiotics for LNB is not included. ^2^CXCL13 was not measured in 17 patients.

### TruCulture®

**Figure 1:**
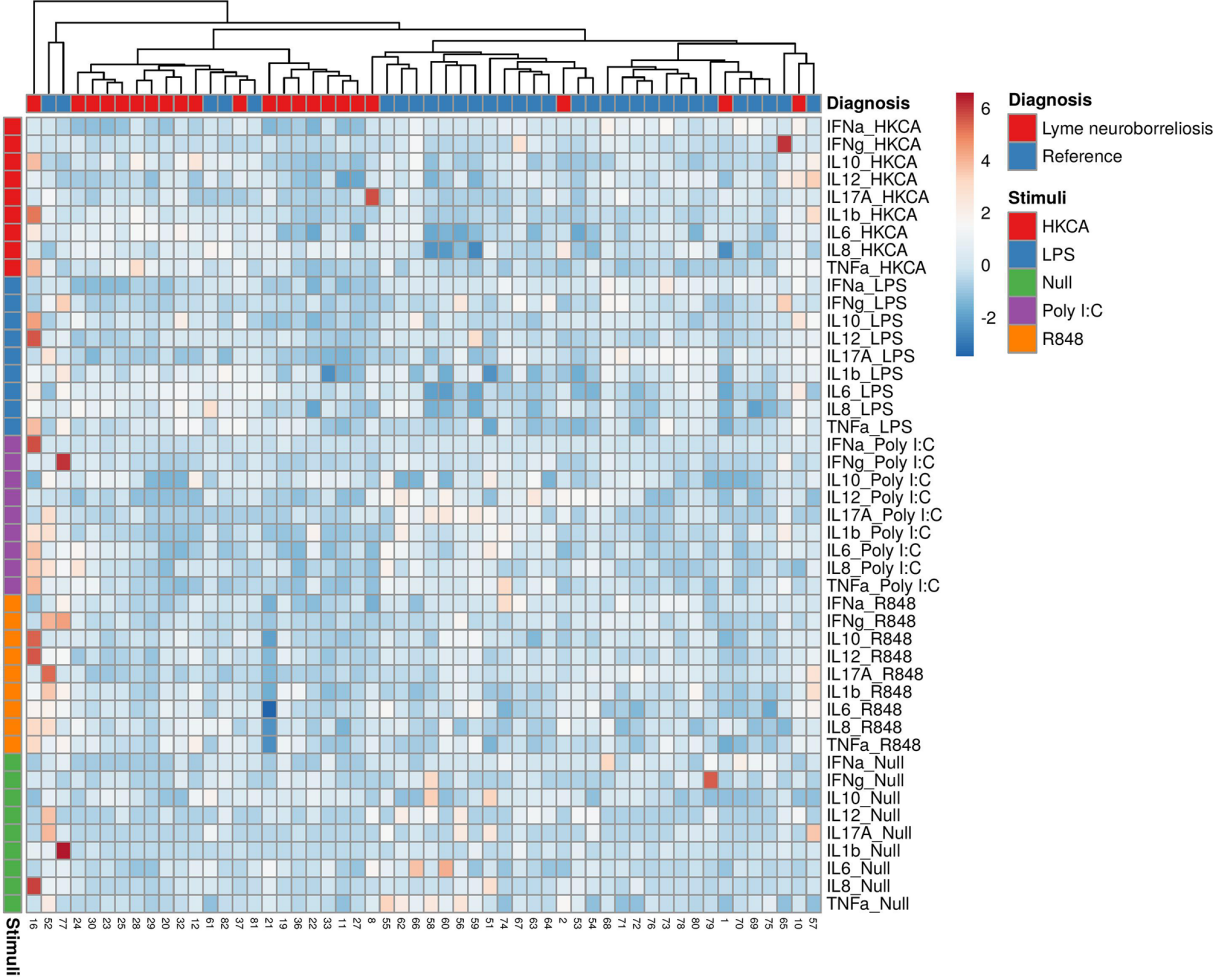
Heatmap of individual cytokine response signatures in patients with LNB (red) and references (blue). The stimulations are divided and shown horizontally, HKCA (red), LPS (blue), PolyI:C (purple), R848 (orange) and Null (green). The individual response is shown in vertical columns and every square represent the cytokine response for the given stimulation. Column (diagnosis) dendrograms were drawn based on hierarchical clustering analysis using the complete agglomeration method on Euclidian distance matrices and Ward linkage.

In general, blood from patients with LNB had similar or slightly decreased concentrations of most cytokines in all TruCulture^®^ stimulations and were clustered with the lowest relative expression compared with references (**Figure 1**).

#### Unstimulated Blood From Patients With LNB Had Lower Concentrations of IFN-α, IL-17A, IL-1β and IL-8 Compared to References

**Figure 2:**
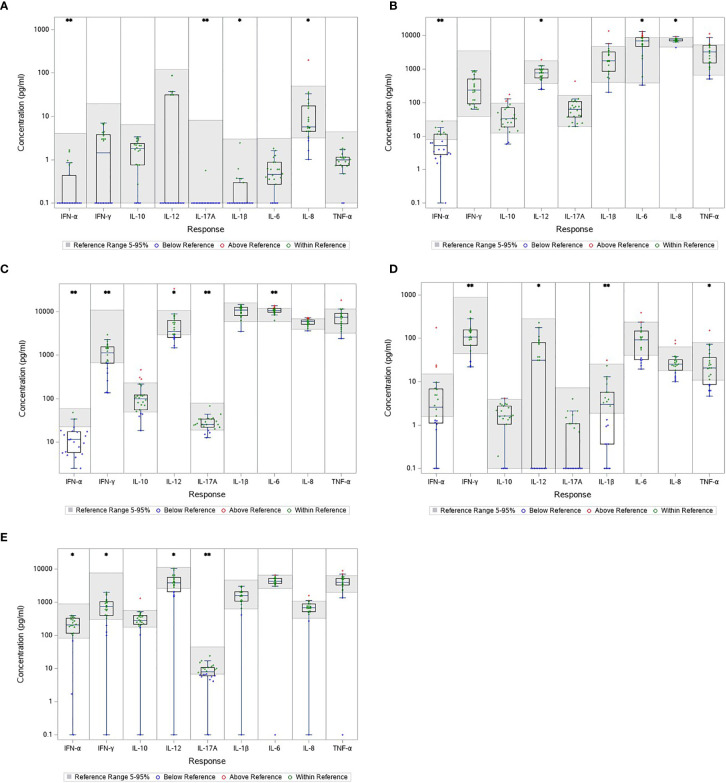
Cytokine concentration in unstimulated **(A)** and stimulated blood samples **(B–E)** from patients with LNB. The gray area represents the reference interval and every concentration is shown individually as color coded dots. Comparison between cytokine concentration in blood from patients with LNB and the reference. *p < 0.05. **p < 0.001. **(A)** NULL **(B)** HCKA **(C)** LPS **(D)** POLY I:C **(E)** R848.

The cytokine levels measured in the unstimulated samples serve as proxy for the circulating cytokine levels and are a crude estimate of the immune response at time of blood sampling. The concentrations of most cytokines in the blood from patients with LNB were comparable with the references, except IFN-α, IL-17A, IL-1β and IL-8 which were significantly below the references (**Figure 2A**). In total, 15 patients (75%) had a concentration of IFN-α below the reference range, 21 patients (95%) had IL-17A concentrations below reference range, 16 patients (73%) had IL-1β concentrations below reference range and 4 (18%) had concentrations of IL-8 below the reference range (**Figure 2A**). The results indicate a slightly lower or suppressed immune response prior to stimulation in patients with LNB.

#### Stimulated Blood From Patients With LNB Had Lower Concentrations of IFN-α, IFN-γ, IL-12 and IL-17A in Multiple Stimulations

TruCulture^®^ was designed to represent a broad spectrum of both intra- and extracellular pathways (R848/Poly I:C and HKCA/LPS, respectively) presenting responses to both fungal, bacterial and viral infections. HKCA stimulate through TLR1/2/4/6, LPS through TLR4, R848 through TLR 7/8 and Poly I:C through TLR3 (Duffy et al., 2014; Duffy et al., 2017). Blood from patients with LNB had similar or slightly decreased concentrations of most cytokines in all stimulations compared with the references. Particular, IFN-α, IFN-γ, IL-12 and IL-17A were significantly lower than the references in multiple stimulations; IFN-α was reduced in HKCA, LPS and R848 stimulated samples with 13(65%), 19(90%) and 3(15%) patients with concentrations below the reference range, respectively (**Figures 2B, C, E**). Concentrations of IFN-γ were below reference in LPS, Poly I:C and R848 stimulated samples and 7(32%), 4(18%) and 4(18%) patients were below the reference range, respectively (**Figures 2C–E**). IL-12 was lower in all the stimulated samples with 2(9%), 8(36%), 10(45%) and 6(27%) having concentrations below reference range in the HKCA, LPS, Poly I:C and R848 stimulated samples respectively (**Figures 2B–E**). IL-17A was lower in LPS and R848 stimulated samples with 4(18%) and 10(45%) having concentrations below reference range (**Figures 2C, E**). These results indicate a less reactive immune response with a low Th1 and Th17 response.

Only two cytokine concentrations were above the references; IL-8 in the HKCA stimulated samples with 1(5%) having concentrations above range, and IL-6 in the LPS and HKCA stimulated samples with 5(23%) and 6(27%) having concentrations above range, respectively. The results suggest a slightly activated immune response to an ongoing CNS infection.

#### Stimulated Blood From Patients With Other CNS Infections Had the Lowest Relative Expression of Cytokines Compared With LNB and References, but in the Unstimulated Blood Samples Higher Concentrations of IL-17A, IL-6 and IL-8 Were Seen

**Figure 3:**
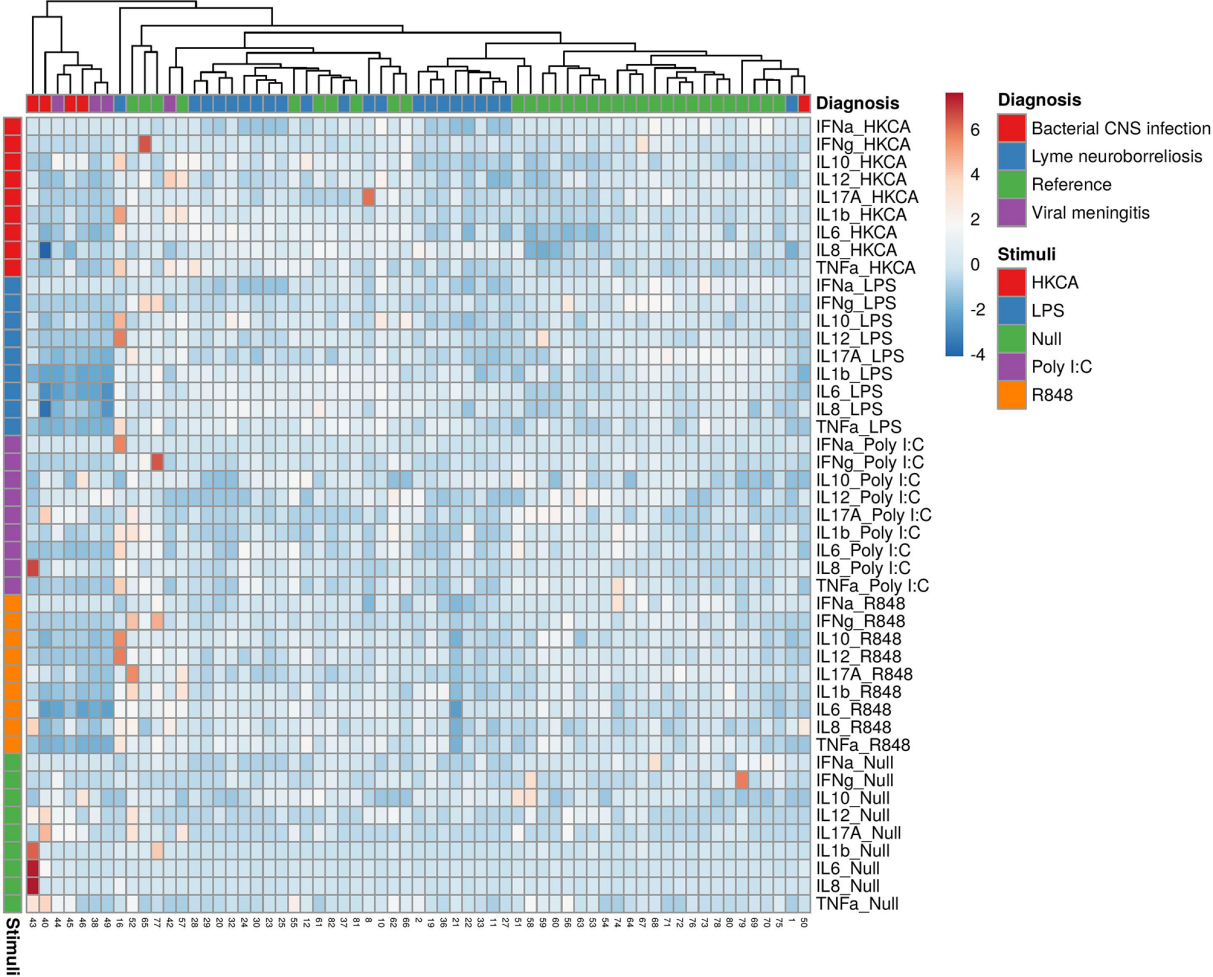
Heatmap of individual cytokine response signatures in blood from patients with LNB (blue), bacterial CNS infections (red), viral meningitis (purple) and references (green). The stimulations are divided and shown horizontally, HKCA (red), LPS (blue), PolyI:C (purple), R848 (orange) and Null (green). The individual response is shown in vertical columns and every square represent the cytokine response for the given stimulation. Column (diagnosis) dendrograms were drawn based on hierarchical clustering analysis using the complete agglomeration method on Euclidian distance matrices and Ward linkage.

**Figure 4:**
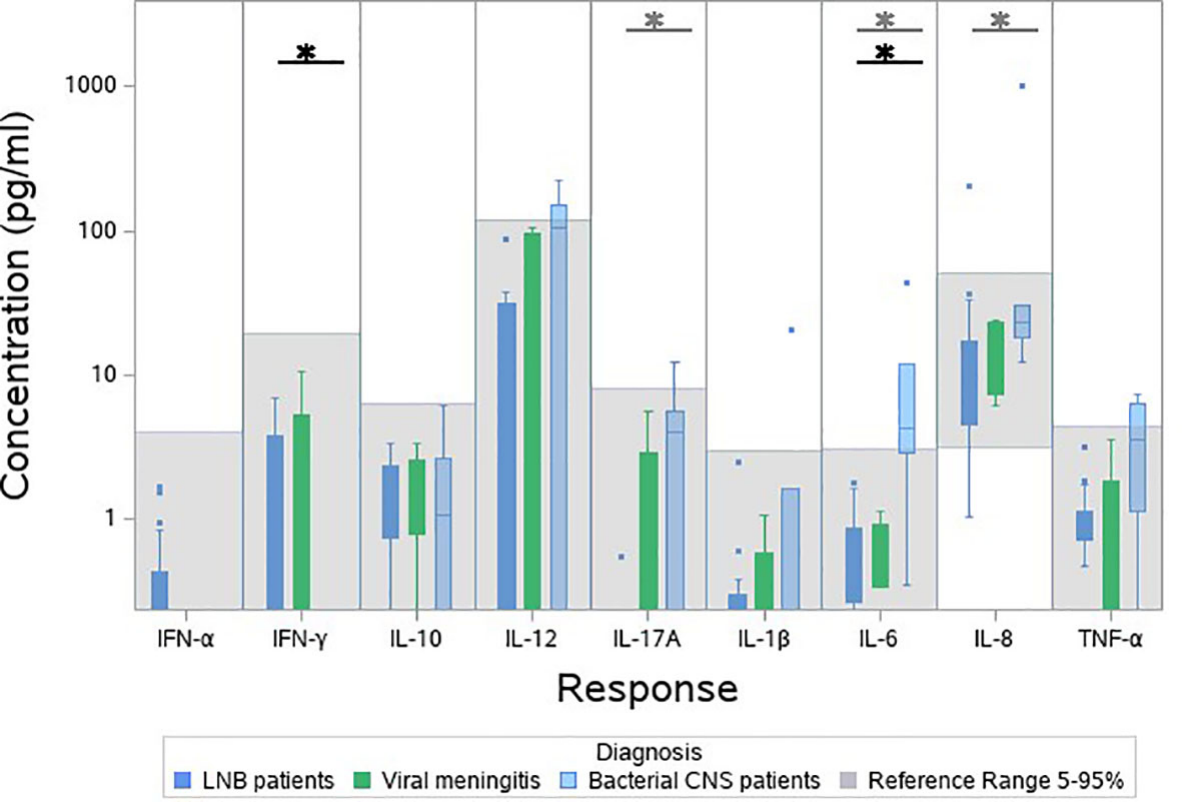
Cytokine concentration in unstimulated blood samples from patients with other bacterial CNS infections (light blue), viral CNS infections (green), patients with LNB (dark blue) and references (grey). Bacterial CNS-infections was compared with LNB (grey stars) and bacterial CNS-infections was compared with references (black stars). *p < 0.05.

As an indication of the responses to the TruCulture^®^ stimulations, we included blood samples from 9 patients with other CNS infections in a post hoc analysis (male/female: 5/4, median age 46.9, IQR 30.1-54.7). An additional table shows the baseline characteristics of patients with other CNS infections (see **Supplementary Table 2**). The patients clustered in groups and other bacterial CNS infections had the lowest relative expression followed by viral CNS infections, LNB and references (**Figure 3**). In the unstimulated samples, other bacterial CNS infections had higher concentrations of both IL-17A, IL-6 and IL-8 compared with patients with LNB and significant higher IL-6 compared with references (**Figure 4**). The findings indicate a reactive and possibly exhausted immune response in patients with other CNS infections.

## Discussion

To our knowledge, this study is the first to investigate the stimulated immune response in blood from patients with LNB using the standardized TruCulture^®^ test-kits. The unstimulated cytokine concentrations in patients with LNB were lower or comparable with references, and the stimulated cytokine release in patients with LNB was slightly lower than the references, indicating a less reactive immune response or impaired signaling pathway. Specific patterns of low IL-12 and IFN-γ were seen across stimulations, indicating a low Th1-response. Low concentrations of IL-17A did not support hyperinflammation or a strong Th17 response. The results indicate that patients with LNB elicit a slightly suppressed or impaired immune response for the investigated stimulations.

The overall finding of reduced expression of cytokines found in patients with LNB compared with references, leave the impression that *B. burgdorferi* may induce immune hyporesponsiveness (**Figure 1**). This was previously demonstrated in human whole blood from patients with late stage LB stimulated with LPS by a decreased TNF-α and IFN-γ response (Diterich et al., 2001). Another hypothesis is that the immune system, when fighting one pathogen, is exhausted and more vulnerable to other pathogens (Diterich et al., 2003). This hypothesis was supported by a German *in vitro* study, where human peripheral blood mononuclear cells (PBMCs) stimulated with *B. burgdorferi* followed by LPS 24h later, presented lower concentrations of TNF-α compared to the PBMCs only stimulated with *B. burgdorferi*. The same pattern was seen in the opposite set-up were the PBMCs were initially stimulated with LPS and later *B. burgdorferi* (Diterich et al., 2003). A double stimulation cannot be ruled out as all patients with LNB, at time of blood sampling, were infected with *B. burgdorferi* and afterwards stimulated with the TruCulture^®^ stimuli. Since spirochetes do not appear for extended duration in blood, and only in low concentrations (Embers et al., 2004; Aguero-Rosenfeld et al., 2005; Pachner and Steiner, 2007), an exhaustion of the immune response in blood from patients with LNB is less likely. However, patients with LNB have typically been infected for 4-6 weeks prior to the symptomatic presentation and some effect cannot be dismissed (Hansen and Lebech, 1992; Mygland et al., 2010). Age has furthermore been linked to a decline in immune response, but also with a general low chronic inflammation with higher concentrations of proinflammatory cytokines (Ray and Yung, 2018). We suspect that patients with LNB had a higher age than the references, which could contribute to a lower stimulated cytokine response. However, we did not find evidence of increased proinflammatory cytokines in the unstimulated samples. Half of the patients (11/22) were treated with Doxycycline for more than 4 days at time of blood sampling. Doxycycline is known to be anti-inflammatory (Bahrami et al., 2012) and shown to inhibit inflammatory responses to *B. burgdorferi* with reductions in TNF-α, IL-6 and IL-8 in an American non-human primate model (Bernardino et al., 2009). It is possible that this may affect our results.

The optimal host mechanism for infection clearance in early LB is a strong Th1-response (Sjöwall et al., 2011), followed by a Th2-response, as an uncontrolled Th1-response seems to increase the symptomatic burden in LB patients (Sjöwall et al., 2005; Shemenski, 2019). Following the tick-bite, the transmission of *B. burgdorferi* is mediated by an inhibition of the Th1-response in favor of a Th2-response primarily by upregulating the immune modulating IL-10 (Shemenski, 2019). We did not find any differences in the concentrations of IL-10 in patients with LNB and references. However, it has been suggested that IL-10 might only be intrathecally produced after dissemination to the CNS (Cepok et al., 2003), which could explain the lack of differences seen in our study.

The four TruCulture^®^ stimulations used in our study represent an activation of different pathways through a variety of TLRs. Interestingly, similar patterns of cytokine responses were found across the stimulations. Particularly, IFN-α, IFN-γ, IL-12 and IL-17A were significantly lower than the references in multiple stimulations. Even in the very different intracellular and extracellular pathway (Duffy et al., 2014; Duffy et al., 2017), decreased concentrations of the four cytokines were found. The phagocytosis of spirochetes seems to be required for IFN-α production and is mediated through TLR7 and TLR9 (Petzke et al., 2009; Cervantes et al., 2014). IFN-α was significantly lower than references in all stimulations except Poly I:C and lower the unstimulated samples. The low expression seen across stimulations could be caused by an exhaustion of the immune response, however due to the low concentration seen in the unstimulated samples, a suppression of the immune response or a defect in the signaling pathway, is more likely. The high missing rate of IFN-α in both references and patients with LNB, however, reduces the power and subsequent interpretation. IL-12 and IFN-γ induce a Th1-response and the production of Th1 T-cells (Cervantes et al., 2014; Shemenski, 2019). IL-12 was found to be significantly increased in whole blood supernatants following stimulation with live *B. burgdorferi* spirochetes in asymptomatic seropositive individuals compared with seronegative controls but not in previous symptomatic seropositive individuals. This finding indicate that IL-12 has a role in optimal eradication of *B. burgdorferi* (Sjöwall et al., 2005). The number of IFN-γ secreting T-cells in PBMC’s was also found to be significantly elevated in active LNB, treated LNB and healthy individuals treated for early LB compared to untreated healthy individuals (van Gorkom et al., 2018). IL-12 and IFN-γ were both reduced in patients with LNB across multiple stimulations, which might indicate a defect or inhibition in the immune system to produce a sufficient Th1-response and to eradicate *B. burgdorferi*.

Studies have suggested a correlation between excessive Th17-response and a clinically unfavorable outcome, possibly due to its hyperinflammatory properties and role in autoimmune diseases (Grygorczuk et al., 2016; Carlsson et al., 2018). Accordingly, a recent Swedish study found subclinical patients with LB to have a lower *B. burgdorferi* stimulated IL-17A compared with clinical LNB patients (Carlsson et al., 2018). Conversely, we found the concentrations of IL-17A to be lower than the references in multiple stimulations, including the unstimulated samples, which does not correspond with a strong Th17-response. Similarly, *Gyllemark et al.* did not find significant differences in serum cytokine response of IL-17A in patients with LNB and healthy controls, but differences were found in CSF, indicating that IL-17A is of intrathecal origin (Gyllemark et al., 2017).

As oppose to the other cytokines, IL-6 was higher in patients with LNB than the references in both LPS and HKCA stimulated samples and IL-8 was increased in HKCA stimulated samples. This do not indicate a defect, but a more prone or slightly activated immune response. IL-8 plays an important role in sustaining inflammation and promoting recruitment of inflammatory cells in the CNS (Grygorczuk et al., 2004; Bernardino et al., 2008). IL-6 induces differentiation of Th17 T-cells as well as the final maturation of B cells into immunoglobulin secreting plasma cells, favoring the secretion of IgG, and it appears to be upregulated in most CNS diseases (Cepok et al., 2003; Bernardino et al., 2008; Tanaka et al., 2014). At time of test, patients with LNB had CSF pleocytosis and most (19/22) also had intrathecal *B. burgdorferi*-antibody production.

The cytokine concentrations were lower or comparable with references in the unstimulated samples. IFN-α, IL-17A, IL-1β and IL-8 were, however, significantly below the references and it seems counterintuitive that the levels of circulating inflammatory cytokines are lower in patients with LNB compared with healthy individuals. It could be an indication of an immune suppression performed by the *B. burgdorferi* infection. Comparing LNB with other bacterial CNS infections in the unstimulated samples, significant lower concentrations of IL-17A, IL-6 and IL-8 were found in patients with LNB (**Figure 4**). This finding corresponds with LNB being a less severe infection and may also partly explain, why common markers for infection such as C-reactive protein (CRP), leucocytes etc. are not increased in patients with LNB (Hansen and Lebech, 1992) but increased in patients with other CNS infections (Julián-Jiménez and Morales-Casado, 2019). Other bacterial CNS infections displayed higher levels of IL-12, IL-17A and TNF-α and statistical higher level of IL-6 compared with references in unstimulated samples, possibly causing an exhaustion of the immune response. This could explain why patients with other CNS infections had the lowest relative expression of cytokines. Patients with LNB had a higher response compared to patients with other CNS infections, but lower than references, this may correspond to a less reactive immune system.

### Strengths and Limitations

Strengths of this study include the well-defined cohort of patients with LNB, where all patients were diagnosed in accordance with the EFNS guidelines. It is so far the first study to investigate the immune response by TruCulture^®^ with a standardized panel of stimulants covering important extra- end intracellular pathways. The study is, however, limited by the small sample size, and the standardized panel lacked the ability to investigate other important pathways such as TLR2 and the adaptive immune response. Though we conducted 5 individual stimulations simultaneously, only one method was used to investigate the immune reaction. Antibiotic treatment was initiated at the time of blood sampling, which most likely reduces inflammation and possibly affects the immune response upon stimulation. Due to the anonymity of the reference group, we did not have access to age or gender, these factors might limit the comparability between groups.

## Conclusion

The cytokine concentrations in unstimulated blood from patients with LNB were lower or comparable with the references. The stimulated cytokine release in blood from patients with Lyme neuroborreliosis was in general slightly lower than in the references. Specific patterns of low IL-12 and IFN-γ were seen across stimulations, indicating a low Th1-response. Low concentrations of IL-17A did not support hyperinflammation or a strong Th17 response. Our results suggest that patients with LNB elicit a slightly suppressed or impaired immune response for the investigated stimulations, however, whether the response normalizes during the course of LNB remains unanswered.

## Data Availability Statement

The raw data supporting the conclusions of this article are available from the corresponding author on reasonable request.

## Ethics Statement

The study protocol and biobank involving human participants were reviewed and approved by the Danish regional ethical committee (Region Hovedstaden)(j.no. H-17024315) and the Danish Data Protection Agency (record no RH-2015-04 I-suite 03605). The participants provided their written informed consent to participate in this study. Baseline characteristics were retrieved from an established quality assessment database.

## Author Contributions

All authors contributed substantially to the design of the study and to the acquisition of data. The statistics was performed by MØ and RG. MØ, RG and A-ML drafted the manuscript. All authors contributed to the article and approved the submitted version.

## Funding

This work was supported as part of NorthTick, an Interreg project supported by the North Sea Programme of the European Regional Development Fund of the European Union, by unrestricted grants from the Augustinus foundation and by PERSIMUNE grant number DNRF126. MØ has received research grant from Rigshospitalets Forskningspuljer. SDN has received research grant from Novo Nordic Foundation.

## Conflict of Interest

Outside of the present work: AM-L reports speakers honorarium/travel grants from Gilead, speakers honorarium/travel grants from GSK, travel grants from MSD and advisory board activity from Gilead. SN reports speakers honorarium/travel grants from Gilead and ViiV/GSK and advisory board activity from Gilead. CS reports speaker honorarium from Boehringer Ingelheim and travel grants from Biogen.

The remaining authors declare that the research was conducted in the absence of any commercial or financial relationships that could be construed as a potential conflict of interest.

## Supplementary Material

Supplementary Figure 1 The TruCulture^®^ set-up. TruCulture^®^ tubes are a closed system for collecting and culturing the whole blood in the presence of customizable stimulants. In this assay, collection tubes are preloaded with the chosen stimulants and culture’s medium. One milliliter of whole blood is drawn directly into the TruCulture^®^ tubes; the tube is incubated for 22 hours. After incubation, the supernatant is separated from cells using a predesigned valve. The concentrations of cytokines and chemokines were measured using 8-plex Luminex. The figure was designed using Gravit Designer.

Supplementary Table 2 Baseline characteristics of 9 patients with other CNS infection at time of blood sample.

## Abbreviations

*B. burgdorferi*, Borrelia burgdorferi sensu lato complex; CNS, Central nervous system; CRP, C-reactive protein; CSF, Cerebrospinal fluid; EFNS, European Federation of Neurological Societies; HKCA, Heat killed Candida albicans; IFN, Interferon; IL, Interleukin; IQR, Interquartile range; LB, Lyme borreliosis; LNB, Lyme neuroborreliosis; LPS, Lipopolysaccharide; Osp, Outer surface protein; PBMC, Peripheral blood mononuclear cells; Poly I:C, Polyinosinic:polycytodylic acid; R848, Resiquimod; TLR, Toll-like receptor.

## References

[B1] Aguero-Rosenfeld M. E. Wang G. Schwartz I. Wormser G. P. (2005). Diagnosis of Lyme Borreliosis. Clin. Microbiol. Rev. 18 (3), 484–509. 10.1128/cmr.18.3.484-509.2005 16020686 PMC1195970

[B2] Bahrami F. Morris D. L. Pourgholami M. H. (2012). Tetracyclines: Drugs With Huge Therapeutic Potential. Mini. Rev. Med. Chem. 12, 44–52. 10.2174/138955712798868977 22070692

[B3] Bernardino A. L. Myers T. A. Alvarez X. Hasegawa A. Philipp M. T. (2008). Toll-Like Receptors: Insights Into Their Possible Role in the Pathogenesis of Lyme Neuroborreliosis. Infect. Immun. 76 (10), 4385–4395. 10.1128/iai.00394-08 18694963 PMC2546821

[B4] Bernardino A. L. Kaushal D. Philipp M. T. (2009). The Antibiotics Doxycycline and Minocycline Inhibit the Inflammatory Responses to the Lyme Disease Spirochete Borrelia Burgdorferi. J. Infect. Dis. 199, 1379–1388. 10.1086/597807 19301981 PMC3697124

[B5] Carlsson H. Ekerfelt C. Henningsson A. J. Brudin L. Tjernberg I. (2018). Subclinical Lyme Borreliosis is Common in South-Eastern Sweden and may be Distinguished From Lyme Neuroborreliosis by Sex, Age and Specific Immune Marker Patterns. Ticks Tick Borne Dis. 9 (3), 742–748. 10.1016/j.ttbdis.2018.02.011 29502989

[B6] Cepok S. Zhou D. Vogel F. Rosche B. Grummel V. Sommer N.. (2003). The Immune Response At Onset and During Recovery From Borrelia Burgdorferi Meningoradiculitis. Arch. Neurol. 60 (6), 849–855. 10.1001/archneur.60.6.849 12810490

[B7] Cervantes J. L. Hawley K. L. Benjamin S. J. Weinerman B. Luu S. M. Salazar J. C. (2014). Phagosomal TLR Signaling Upon Borrelia Burgdorferi Infection. Front. Cell Infect. Microbiol. 4, 55. 10.3389/fcimb.2014.00055 24904837 PMC4033037

[B8] Cervantes J. L. La Vake C. J. Weinerman B. Luu S. O’Connell C. Verardi P. H.. (2013). Human TLR8 is Activated Upon Recognition of Borrelia Burgdorferi RNA in the Phagosome of Human Monocytes. J. Leukoc. Biol. 94 (6), 1231–1241. 10.1189/jlb.0413206 23906644 PMC3828603

[B9] Cutler S. Vayssier-Taussat M. Estrada-Peña A. Potkonjak A. Mihalca A. D. Zeller H. (2019). A New Borrelia on the Block: Borrelia Miyamotoi - a Human Health Risk? Euro. Surveill. 24 (18). 10.2807/1560-7917.es.2019.24.18.1800170 PMC6505184 31064634

[B10] Diterich I. Härter L. Hassler D. Wendel A. Hartung T. (2001). Modulation of Cytokine Release in Ex Vivo-Stimulated Blood From Borreliosis Patients. Infect. Immun. 69 (2), 687–694. 10.1128/iai.69.2.687-694.2001 11159956 PMC97940

[B11] Diterich I. Rauter C. Kirschning C. J. Hartung T. (2003). Borrelia Burgdorferi-Induced Tolerance as a Model of Persistence Via Immunosuppression. Infect. Immun. 71 (7), 3979–3987. 10.1128/iai.71.7.3979-3987.2003 12819085 PMC162029

[B12] Dudek S. Ziółko E. Kimsa-Dudek M. Solarz K. Mazurek U. Wierzgoń A.. (2017). Expression Profiles of Toll-Like Receptors in the Differentiation of an Infection With Borrelia Burgdorferi Sensu Lato Spirochetes. Arch. Immunol. Ther. Exp. (Warsz). 65 (2), 175–182. 10.1007/s00005-016-0416-8 27604757

[B13] Duffy D. Rouilly V. Braudeau C. Corbière V. Djebali R. Ungeheuer M. N.. (2017). Standardized Whole Blood Stimulation Improves Immunomonitoring of Induced Immune Responses in Multi-Center Study. Clin. Immunol. 183, 325–335. 10.1016/j.clim.2017.09.019 28943400

[B14] Duffy D. Rouilly V. Libri V. Hasan M. Beitz B. David M.. (2014). Functional Analysis Via Standardized Whole-Blood Stimulation Systems Defines the Boundaries of a Healthy Immune Response to Complex Stimuli. Immunity 40 (3), 436–450. 10.1016/j.immuni.2014.03.002 24656047

[B15] Embers M. E. Ramamoorthy R. Philipp M. T. (2004). Survival Strategies of Borrelia Burgdorferi, the Etiologic Agent of Lyme Disease. Microbes Infect. 6 (3), 312–318. 10.1016/j.micinf.2003.11.014 15065567

[B16] Grygorczuk S. Świerzbińska R. Moniuszko A. Kondrusik M. Zajkowska J. Czupryna P.. (2016). Synthesis of Th17 Cytokines in the Culture of Peripheral Blood Mononuclear Cells Stimulated With Borrelia Burgdorferi Sensu Lato. Ann. Agric. Environ. Med. 23 (2), 242–247. 10.5604/12321966.1203884 27294626

[B17] Grygorczuk S. Pancewicz S. Zajkowska J. Kondrusik M. Rwierzbińska R. Hermanowska-Szpakowicz T. (2004). Concentrations of Macrophage Inflammatory Proteins MIP-1alpha and MIP-1beta and Interleukin 8 (il-8) in Lyme Borreliosis. Infection 32 (6), 350–355. 10.1007/s15010-004-3110-4 15597225

[B18] Gyllemark P. Forsberg P. Ernerudh J. Henningsson A. J. (2017). Intrathecal Th17- and B Cell-Associated Cytokine and Chemokine Responses in Relation to Clinical Outcome in Lyme Neuroborreliosis: A Large Retrospective Study. J. Neuroinflamm. 14 (1), 27. 10.1186/s12974-017-0789-6 PMC5286657 28148307

[B19] Hansen K. Crone C. Kristoferitsch W. (2013). Lyme Neuroborreliosis. Handb. Clin. Neurol. 115, 559–575. 10.1016/b978-0-444-52902-2.00032-1 23931802

[B20] Hansen K. Lebech A. M. (1992). The Clinical and Epidemiological Profile of Lyme Neuroborreliosis in Denmark 1985-1990. A Prospective Study of 187 Patients With Borrelia Burgdorferi Specific Intrathecal Antibody Production. Brain 115 (Pt 2), 399–423. 10.1093/brain/115.2.399 1606475

[B21] Julián-Jiménez A. Morales-Casado M. I. (2019). Usefulness of Blood and Cerebrospinal Fluid Laboratory Testing to Predict Bacterial Meningitis in the Emergency Department. Neurologia 34 (2), 105–113. 10.1016/j.nrl.2016.05.009 27469578

[B22] Kruger H. Reuss K. Pulz M. Rohrbach E. Pflughaupt K. W. Martin R.. (1989). Meningoradiculitis and Encephalomyelitis Due to Borrelia Burgdorferi: A Follow-Up Study of 72 Patients Over 27 Years. J. Neurol. 236 (6), 322–328. 10.1007/bf00314373 2795099

[B23] Margos G. Fedorova N. Becker N. S. Kleinjan J. E. Marosevic D. Krebs S.. (2020). Borrelia Maritima Sp. Nov., a Novel Species of the Borrelia Burgdorferi Sensu Lato Complex, Occupying a Basal Position to North American Species. Int. J. Syst. Evol. Microbiol. 70 (2), 849–856. 10.1099/ijsem.0.003833 31793856

[B24] Mygland A. Ljøstad U. Fingerle V. Rupprecht T. Schmutzhard E. Steiner I. (2010). EFNS Guidelines on the Diagnosis and Management of European Lyme Neuroborreliosis. Eur. J. Neurol. 17 (1), 8–16.e11–14. 10.1111/j.1468-1331.2009.02862.x 19930447

[B25] Oosting M. Berende A. Sturm P. Ter Hofstede H. J. de Jong D. J. Kanneganti T. D.. (2010). Recognition of Borrelia Burgdorferi by NOD2 is Central for the Induction of an Inflammatory Reaction. J. Infect. Dis. 201 (12), 1849–1858. 10.1086/652871 20441518

[B26] Oosting M. Buffen K. van der Meer J. W. Netea M. G. Joosten L. A. (2016). Innate Immunity Networks During Infection With Borrelia Burgdorferi. Crit. Rev. Microbiol. 42 (2), 233–244. 10.3109/1040841x.2014.929563 24963691

[B27] Ozinsky A. Underhill D. M. Fontenot J. D. Hajjar A. M. Smith K. D. Wilson C. B.. (2000). The Repertoire for Pattern Recognition of Pathogens by the Innate Immune System is Defined by Cooperation Between Toll-Like Receptors. Proc. Natl. Acad. Sci. U. S. A. 97 (25), 13766–13771. 10.1073/pnas.250476497 11095740 PMC17650

[B28] Pachner A. R. Steiner I. (2007). Lyme Neuroborreliosis: Infection, Immunity, and Inflammation. Lancet Neurol. 6 (6), 544–552. 10.1016/s1474-4422(07)70128-x 17509489

[B29] Petnicki-Ocwieja T. Kern A. (2014). Mechanisms of Borrelia Burgdorferi Internalization and Intracellular Innate Immune Signaling. Front. Cell Infect. Microbiol. 4, 175. 10.3389/fcimb.2014.00175 25566512 PMC4266086

[B30] Petzke M. M. Brooks A. Krupna M. A. Mordue D. Schwartz I. (2009). Recognition of Borrelia Burgdorferi, the Lyme Disease Spirochete, by TLR7 and TLR9 Induces a Type I IFN Response by Human Immune Cells. J. Immunol. 183 (8), 5279–5292. 10.4049/jimmunol.0901390 19794067

[B31] Petzke M. Schwartz I. (2015). Borrelia Burgdorferi Pathogenesis and the Immune Response. Clin. Lab. Med. 35 (4), 745–764. 10.1016/j.cll.2015.07.004 26593255

[B32] Ray D. Yung R. (2018). Immune Senescence, Epigenetics and Autoimmunity. Clin. Immunol. 196, 59–63. 10.1016/j.clim.2018.04.002 29654845 PMC6548177

[B33] Shemenski J. (2019). Cimetidine as a Novel Adjunctive Treatment for Early Stage Lyme Disease. Med. Hypotheses 128, 94–100. 10.1016/j.mehy.2016.03.015 27107653

[B34] Sjöwall J. Carlsson A. Vaarala O. Bergström S. Ernerudh J. Forsberg P.. (2005). Innate Immune Responses in Lyme Borreliosis: Enhanced Tumour Necrosis Factor-Alpha and interleukin-12 in Asymptomatic Individuals in Response to Live Spirochetes. Clin. Exp. Immunol. 141 (1), 89–98. 10.1111/j.1365-2249.2005.02820.x 15958074 PMC1809414

[B35] Sjöwall J. Fryland L. Nordberg M. Sjögren F. Garpmo U. Jansson C.. (2011). Decreased Th1-type Inflammatory Cytokine Expression in the Skin is Associated With Persisting Symptoms After Treatment of Erythema Migrans. PloS One 6 (3), e18220. 10.1371/journal.pone.0018220 21483819 PMC3069060

[B36] Stanek G. Wormser G. P. Gray J. Strle F. (2012). Lyme Borreliosis. Lancet 379 (9814), 461–473. 10.1016/s0140-6736(11)60103-7 21903253

[B37] Steere A. C. Strle F. Wormser G. P. Hu L. T. Branda J. A. Hovius J. W.. (2016). Lyme Borreliosis. Nat. Rev. Dis. Primers 2, 16090. 10.1038/nrdp.2016.90 27976670 PMC5539539

[B38] Tanaka T. Narazaki M. Kishimoto T. (2014). IL-6 in Inflammation, Immunity, and Disease. Cold Spring Harb. Perspect. Biol. 6 (10), a016295. 10.1101/cshperspect.a016295 25190079 PMC4176007

[B39] van Gorkom T. Sankatsing S. U. C. Voet W. Ismail D. M. Muilwijk R. H. Salomons M.. (2018). An Enzyme-Linked Immunosorbent Spot Assay Measuring Borrelia Burgdorferi B31-Specific Interferon Gamma-Secreting T Cells Cannot Discriminate Active Lyme Neuroborreliosis From Past Lyme Borreliosis: A Prospective Study in the Netherlands. J. Clin. Microbiol. 56 (4), e01695–17. 10.1128/jcm.01695-17 PMC5869815 29367297

[B40] Waindok P. Schicht S. Fingerle V. Strube C. (2017). Lyme Borreliae Prevalence and Genospecies Distribution in Ticks Removed From Humans. Ticks Tick Borne Diseases 8 (5), 709–714. 10.1016/j.ttbdis.2017.05.003 28528880

