# Supplementary figures and images for: Stimulated Immune Response by TruCulture® Whole Blood Assay in Patients With European Lyme Neuroborreliosis: A Prospective Cohort Study
Source: Front Cell Infect Microbiol. 2021 May 10;11:666037. doi: 10.3389/fcimb.2021.666037 (PMC8141554; doi:10.3389/fcimb.2021.666037)

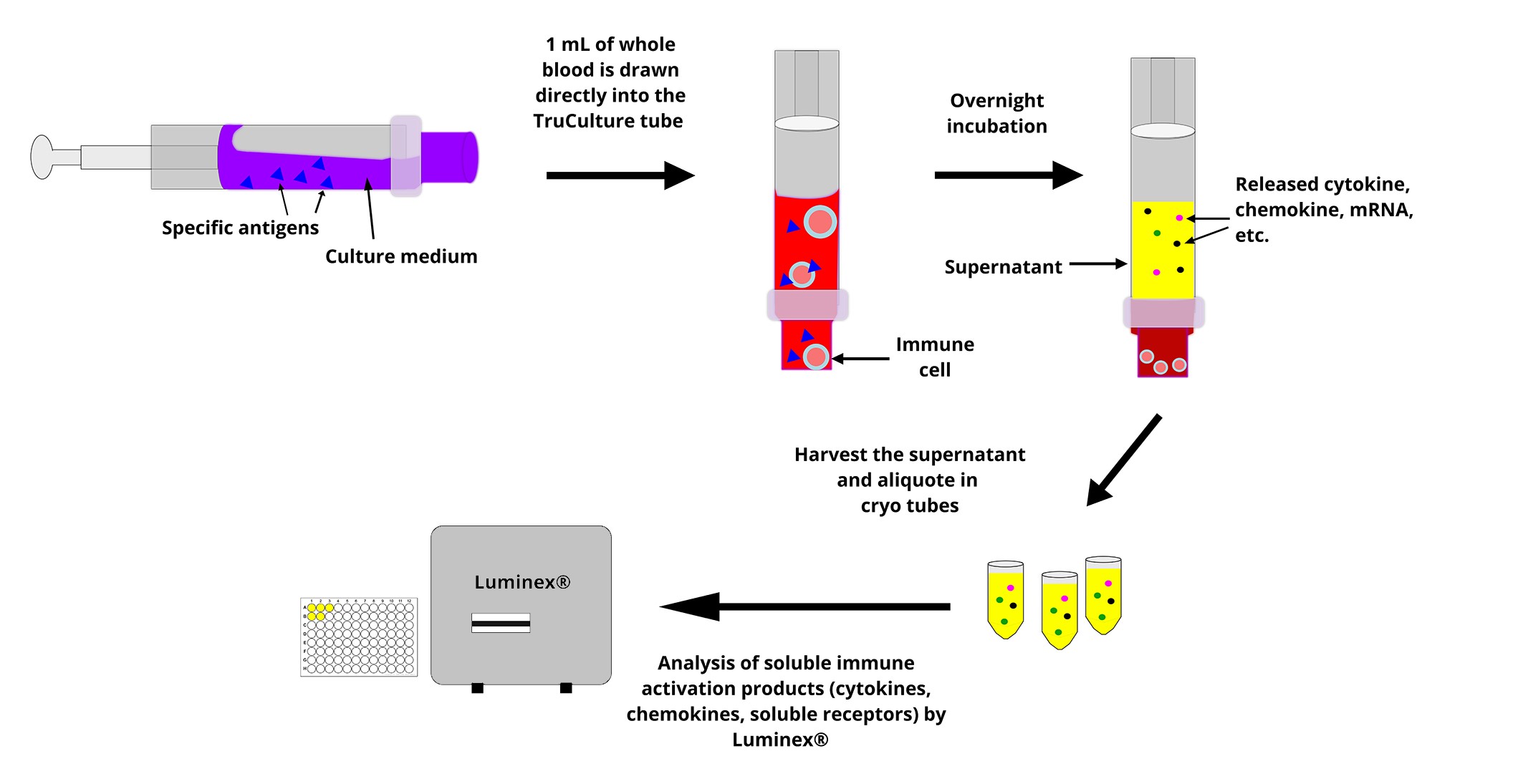

Supplement: Supplementary Figure 1 — The TruCulture® set-up. TruCulture® tubes are a closed system for collecting and culturing the whole blood in the presence of customizable stimulants. In this assay, collection tubes are preloaded with the chosen stimulants and culture’s medium. One milliliter of whole blood is drawn directly into the TruCulture® tubes; the tube is incubated for 22 hours. After incubation, the supernatant is separated from cells using a predesigned valve. The concentrations of cytokines and chemokines were measured using 8-plex Luminex. The figure was designed using Gravit Designer. [file Image_1.jpeg]
