# Supplementary material for: Stimulated Immune Response by TruCulture® Whole Blood Assay in Patients With European Lyme Neuroborreliosis: A Prospective Cohort Study
Source: Front Cell Infect Microbiol. 2021 May 10;11:666037. doi: 10.3389/fcimb.2021.666037 (PMC8141554; doi:10.3389/fcimb.2021.666037)
Supplement: Supplementary Table 2 — Baseline characteristics of 9 patients with other CNS infection at time of blood sample. [file Table_1.docx]

| Table 1 Baseline characteristics of patients with other CNS infection at time of blood sample | |
| --- | --- |
|  | Other CNS infections  (n=9) |
| Age, median (IQR) | 46 (30-54) |
| Male, n (%) | 5 (56) |
| No comorbidities, n (%) | 4 (44) |
| No prescription medication^1^, n (%) | 4 (44) |
| Durations of symptoms, median (IQR) | 4 (2-24) |
| Diagnosis | |
| Viral CNS infection |  |
| Enteroviral meningitis | 2 (23) |
| Herpes simplex viral meningitis | 1 (11) |
| Unidentified viral meningitis | 1 (11) |
| Bacterial CNS infection |  |
| Unidentified bacterial meningitis | 1 (11) |
| Unidentified bacterial encephalitis | 1 (11) |
| Cerebral abscess |  |
| Staphylococcus aureus^2^ | 1 (11) |
| Staphylococcus epidermidis | 1 (11) |
| Pseudomonas aeruginosa | 1 (11) |
| Laboratory results | |
| Serum leucocytes, median (IQR) | 9.5 (5.8 – 10.0) |
| Neutrophils, median (IQR) | 6.4 (4.5 – 8.3) |
| CSF pleocytosis, median (IQR) | 515 (224-785) |
| Abbreviations: IQR, Interquartile range; CSF, Cerebrospinal fluid;  Categorical variables are presented as n (%) and continuous variables as medians with interquartile rates (IQRs).  ^1^ Other than antibiotics. ^2^ Septic-embolic brain abscesses | |
